# HGF/c-MET axis contributes to CLL cell survival by regulating multiple mechanisms making it a potential therapeutic target for CLL treatment

**DOI:** 10.3389/fphar.2025.1612916

**Published:** 2025-05-30

**Authors:** Shihao Liang, Xiaoya Shao, Xueqiong Meng, Ying Cui, Chuanyue Sun, Jie Sun, Binghui Zhang, Guomin Shen, Ling Qin, Haiping Yang, Yixiang Chen

**Affiliations:** ^1^ Henan International Joint Laboratory of Thrombosis and Hemostasis, School of Basic Medical Sciences, Henan University of Science and Technology, Luoyang, China; ^2^ The Second Affiliated Hospital, Henan University of Science and Technology, Luoyang, China; ^3^ Luoyang Key Laboratory of POCT Diagnostic Technology, Luoyang Polytechnic, Luoyang, China; ^4^ Zhongyuan Scholars Workstation of Henan, Luoyang Polytechnic, Luoyang, China; ^5^ Henan Province Engineering Technology Research Center of Key Immunological Biomaterials, Luoyang Polytechnic, Luoyang, China; ^6^ The First Affiliated Hospital, Henan University of Science and Technology, Luoyang, China

**Keywords:** chronic lymphocytic leukemia, HGF/c-Met, signaling pathway, anti-apoptotic proteins, capmatinib

## Abstract

Despite significant advances in understanding the occurrence, progression and treatment of chronic lymphocytic leukemia (CLL), there remains a need to explore novel mechanisms and therapeutic strategies. In this study, we discovered that hepatocyte growth factor (HGF), a cytokine highly expressed by bone marrow mesenchymal stem cells within the microenvironment, activates the AKT, ERK and STAT3 signaling pathways, promotes the expression of anti-apoptotic proteins BCL-2, MCL-1, and BCL-xL, thereby enhancing CLL cell survival and resistance to both natural and ABT-199-induced apoptosis. Knockdown of c-MET, the unique receptor for HGF, using lentivirus-mediated shRNA, significantly attenuated the activation of these pro-survival signaling pathways and downregulated the expression of anti-apoptotic proteins in the BCL-2 family. Consequently, this inhibited CLL cell proliferation and promoted apoptosis. Similarly, pharmacological targeting of the HGF/c-MET pathway with the inhibitor capmatinib markedly suppressed the activation of pro-survival signaling pathways, reduced the expression of anti-apoptotic proteins, inhibited cell proliferation, arrested cell cycle at G0/G1 stage, induced apoptosis, and enhanced the pro-apoptotic effect of ABT-199. In summary, this study highlights the critical role of HGF/c-MET axis in CLL cell survival and demonstrates that targeting this pathway holds therapeutic potential for the treatment of CLL.

## 1 Introduction

Chronic lymphocytic leukemia (CLL), the most common adult leukemia in Western countries, arises from clonal expansion of CD5+/CD19+ mature B-cells in the blood, bone marrow, and lymphoid tissues due to disrupted apoptosis and proliferation mechanisms (Chiorazzi et al., 2021). The pathogenesis of CLL is driven by multiple factors such as the genetic mutations, chromosomal abnormalities, constitutive activation of B-Cell Receptor (BCR) Signaling, dynamic interaction between CLL cells and the tumor microenvironment, and the overexpression of anti-apoptotic BCL-2 family proteins (Chiorazzi et al., 2021; Chen et al., 2018). Although targeted therapies, such as BCL2 inhibitors, have markedly improved outcomes, with prolonging progression-free survival and overall survival across risk groups (Patel and Pagel, 2021; Stumpf and Al-Sawaf, 2024). CLL remains incurable in most cases, and significant challenges persist. Consequently, there is a pressing need to explore novel pathogenic mechanisms and therapeutic strategies particular the innovative drug combinations.

Hepatocyte growth factor (HGF), also known as scatter factor, is the natural ligand of the c-MET (mesenchymal-epithelial transition tyrosine kinase receptor). Under physiological conditions, HGF is predominantly produced by bone marrow stromal cells (BMSC) (Bladt et al., 1995; Bussolino et al., 1992; Huh et al., 2004). As a multifunctional growth factor, HGF interacts with c-MET to regulate diverse physiological processes, including embryonic development, wound healing, angiogenesis, tissue regeneration and organ morphogenesis (Trusolino et al., 2010). However, dysregulated activation of HGF/c-MET axis contributes to the development and progression of numerous human cancers, such as gastroesophageal adenocarcinoma, cholangiocarcinoma, colon cancer, renal cell carcinoma, glioblastomas, and lung cancer, among others (Catenacci et al., 2017; Heo et al., 2017; Gayyed et al., 2015; Peltola et al., 2017). The axis mediates activation of downstream signaling pathways that regulate critical biological processes, including cell proliferation, survival, inhibition of apoptosis, migration, invasion and metastasis (Brown, 2016; Hofbauer et al., 2010; Giannoni et al., 2011; Cascone et al., 2017; Ogunwobi et al., 2013; Ono et al., 2006). Aberrant HGF/c-MET signaling is also strongly associated with poor clinical outcomes and drug resistance (Mahtouk et al., 2010; Guo et al., 2020). While the role of HGF/c-MET axis activation has been extensively studied in solid tumors, its potential in hematological malignancies remains underexplored.

In acute myelocytic leukemia (AML) and multiple myeloma (MM), elevated plasma HGF levels have been identified as a prognostic factor (Verstovsek et al., 2001; Gambella et al., 2015). Similarly, in diffuse large B cell lymphoma (DLBCL), patients demonstrated increased HGF and c-MET in their blood, bone marrow, plasma, and pleural fluid, which correlates with clinic outcomes (Tjin et al., 2006). In CLL patients, serum HGF levels are significantly higher compared to the healthy controls (Eksioglu-Demiralp et al., 2011). Notably, within the bone marrow (BM) and lymph-nodes of CLL patients, cells morphologically resembling nurse-like cells (NLCs) frequently exhibit c-MET positive (Giannoni et al., 2014). While prior studies report that HGF/c-MET interaction activates STAT3 signaling pathway and promotes the survival of CLL cells (Giannoni et al., 2011), the precise mechanistic details underlying pro-survival effect remain to be fully elucidated.

In this study, we explored the effects and underlying mechanisms of HGF/c-MET axis in CLL. We found that besides the STAT3 signaling pathway, the HGF/c-MET axis also triggers pro-survival AKT and ERK signaling pathways and upregulates the expression of multiple anti-apoptotic BCL-2 family proteins, thereby promoting cell survival and resistance to ABT-199 (venetoclax). Genetic knockdown of the c-MET gene via lentivirus-delivered shRNA, as well as pharmacological inhibition of HGF/c-MET pathway using the small-molecule inhibitor capmatinib, attenuated these pro-survival signals and effectively induced apoptosis in CLL cells. Strikingly, combing capmatinib with ABT-199 synergistically enhanced apoptosis, highlighting the potential therapeutic application in CLL treatment.

## 2 Materials and methods

### 2.1 Cell culture and reagents

The MEC-1 cell line, a chronic B-cell leukemia cell was cultured in RPMI-1640 medium (Corning) supplemented with 10% (v/v) fetal bovine serum (FBS) (ABW company) and 50U/ml penicillin, 50 mg/mL streptomycin (Solarbio) at 37°C incubator containing 5% CO_2_. Peripheral blood samples were collected from CLL patients at the First Affiliated Hospital of Henan University of Science and Technology, following informed consent and approval from the local research ethics committee. (Supplementary Table S1). Primary CLL cells were isolated using STEMCELL 19,664 B-CLL Sorting Kit (EasySep™ Direct Human B-CLL Cell Isolation Kit, Canada) and cultured in RPMI-1640 medium supplemented with 10 ng/mL CD40L and IL-4 (Peprotech). HGF was obtained from (Proteintech). Capmatinib and ABT-199 were purchased from MedChemExpress (MCE). All inhibitors were dissolved in dimethyl sulfoxide (DMSO) form experimental use.

### 2.2 Construction of c-MET-shRNA lentivirus

To establish a lentiviral transfection system targeting the HGF receptor c-MET, the following steps were carried out: Three 97 bp shRNA sequences targeting c-MET (shRNA-c-MET) were designed based on the classical reference sequence NM_000245.4 using the online SplashRNA software (http://splashrna.mskcc.org) (Pelossof et al., 2017) (Supplementary Table S2). The 163 bp shRNA-c-MET fragments were then amplified by PCR using the synthesized primers (Supplementary Table S3). These fragments were subsequently inserted into the lentiviral vector plasmid Lenti-TRE-TurboRFP-miR-shRNA-zeocin. The constructed plasmid was validated through XhoI/EcoRI double enzyme digestion and sequencing. The validated vector plasmid, along with the packaging plasmid psPAX2 and the envelope plasmid pMD2.G, was co-transfected into HEK293T cells. The cell culture supernatant containing lentiviral particles was collected. MEC-1 cells were infected with the collected lentiviral particles and induced with 1 μg/mL doxycycline (Dox). Transfection efficiency was assessed using immunofluorescence microscopy. After 72 h, the transfected MEC-1 cells were harvested for subsequent experiments.

### 2.3 Cell proliferation assays (MTS)

Cell proliferation was evaluated by the CellTiter 96 Aqueous One Solution Cell Proliferation MTS Assay (Promega), following the manufacturer’s instructions as previously described (Chen et al., 2016). Briefly, cells were seeded in 96-well plates, and after treatment, 20 µL of MTS reagent was added to each well. The plates were incubated at 37°C for 1–4 h, and the absorbance was measured at 490 nm using a microplate reader (ALLSheng). Each experiment was performed in triplicate and repeated at least three times. Relative cell viability (%) was calculated by comparing the absorbance values of treated cells to those of untreated control cells.

### 2.4 Assessment of cell apoptosis and cell cycle analysis

Cell apoptosis was assessed using an Annexin-V-propidium iodide (PI) apoptosis detection kit (Biolegend) following 48 h of treatment. Cells were harvested, washed with PBS, and stained with 5 µL Annexin V-FITC and 10 µL PI solution for 15 min at room temperature (25°C) in the dark. The percentage of apoptotic cells was determined by flow cytometry using a FACS Canto II cytometer (BD Biosciences), Data were analyzed using FlowJo software. Each experiment was performed in duplicate and repeated three independent times. For cell cycle analysis, cells were harvested, washed with PBS, and fixed with cold 70% ethanol for at least 2 h at 4°C or −20°C. After fixation, cells were washed with PBS and treated with 0.2–0.5 μg/mL RNase (Solarbio) at 37°C for 30 min in the dark. Cells were then stained with 10 μg/mL PI solution and incubated at 37°C for 30 min in the dark. Cell cycle distribution was analyzed using a FACS Canto II cytometer (BD Biosciences), and data were processed using ModFit LT software. Experiment was performed in triplicate and repeated at least three time.

### 2.5 Western blotting

The whole-cell proteins were lysed using SDS sample buffer (Solarbio) containing 1% protease inhibitor cocktail and 1% phosphatase inhibitor cocktail (Sigma). Protein samples were boiled for 5–10 min and separated by SDS-PAGE at 120 V for 1.5 h. The separated proteins were then transferred to nitrocellulose (NC) membranes (GE Healthcare) at 0.35 A for 1.5 h in the cold room. The NC membranes were blocked with 5% (w/v) bovine serum albumin (BSA) in PBS containing 0.05% Tween-20 for 1 h at room temperature. Subsequently, the membranes were incubated with specific primary antibodies (diluted 1:1,000) either at room temperature for 1 h or at 4°C overnight. After washing, the membranes were incubated with horseradish peroxidase (HRP)-conjugated secondary antibodies (diluted 1:5,000) for 1 h at room temperature. The following primary antibodies were used: AKT, phospho-AKT (Ser473), ERK, phospho-ERK(Thr202/Tyr204), STAT3, p-STAT3 (Tyr705), caspase-3 and PARP (Cell Signaling Technology, CST); BCL-xL (Solarbio); MCL-1 and p-MET (F-5) (Santa Cruz); HGF, c-MET, and cleaved-caspase-3 (Proteintech); BAX, anti-rabbit, and anti-mouse secondary antibodies (Servicebio); BCL-2 and β-actin (Bioss). After incubation with the ECL luminescent solution (Applygen) according to the manufacturer’s instructions, and protein bands were visualized in a chemiluminescence detector (Tianneng) and quantified by ImageJ software.

### 2.6 Quantitative Real-Time PCR (qRT-PCR)

Total RNA was extracted using Eastep™ Super Total RNA Extraction Kit (Promega), and cDNA was synthesized using cDNA Synthesis SuperMix (Novoprotein). Quantitative PCR (qPCR) was performed on ABI Prism^®^ 7,500 Real-Time PCR System (ABI, United States) using SYBR-Green qPCR Master Mix (Low Rox) (MCE) following the manufacturer’s instructions. Each reactions mixture (20 µL) contained 10 µL of SYBR Green Master Mix (2×), 0.2 µM of each forward and reverse primer and 0.5–10 ng/μL cDNA. The primer sequences used are listed in Supplementary Table S4. The PCR conditions were as follows: initial denaturation at 95°C for 5 min, followed by 40 cycles of denaturation at 95°C for 20 s and annealing/extension at 60°C for 40 s. Relative gene expression was calculated using the 2^−ΔΔCT^ method (Livak and Schmittgen, 2001), with β-actin as the internal control housekeeping gene. All experiments were performed in triplicate and repeated at least three times.

### 2.7 Statistical analysis

Data were presented as Mean ± SD or SEM. Statistical analyses were conducted using GraphPad Prism 8.0. Differences between treatments groups were assessed using Student’s t-test or two-way ANOVA, as appropriate. A *p*-value of <0.05 was considered as statistical significance.

## 3 Results

### 3.1 HGF promotes CLL cells survival and confers resistance to ABT-199-induced apoptosis

**FIGURE 1:**
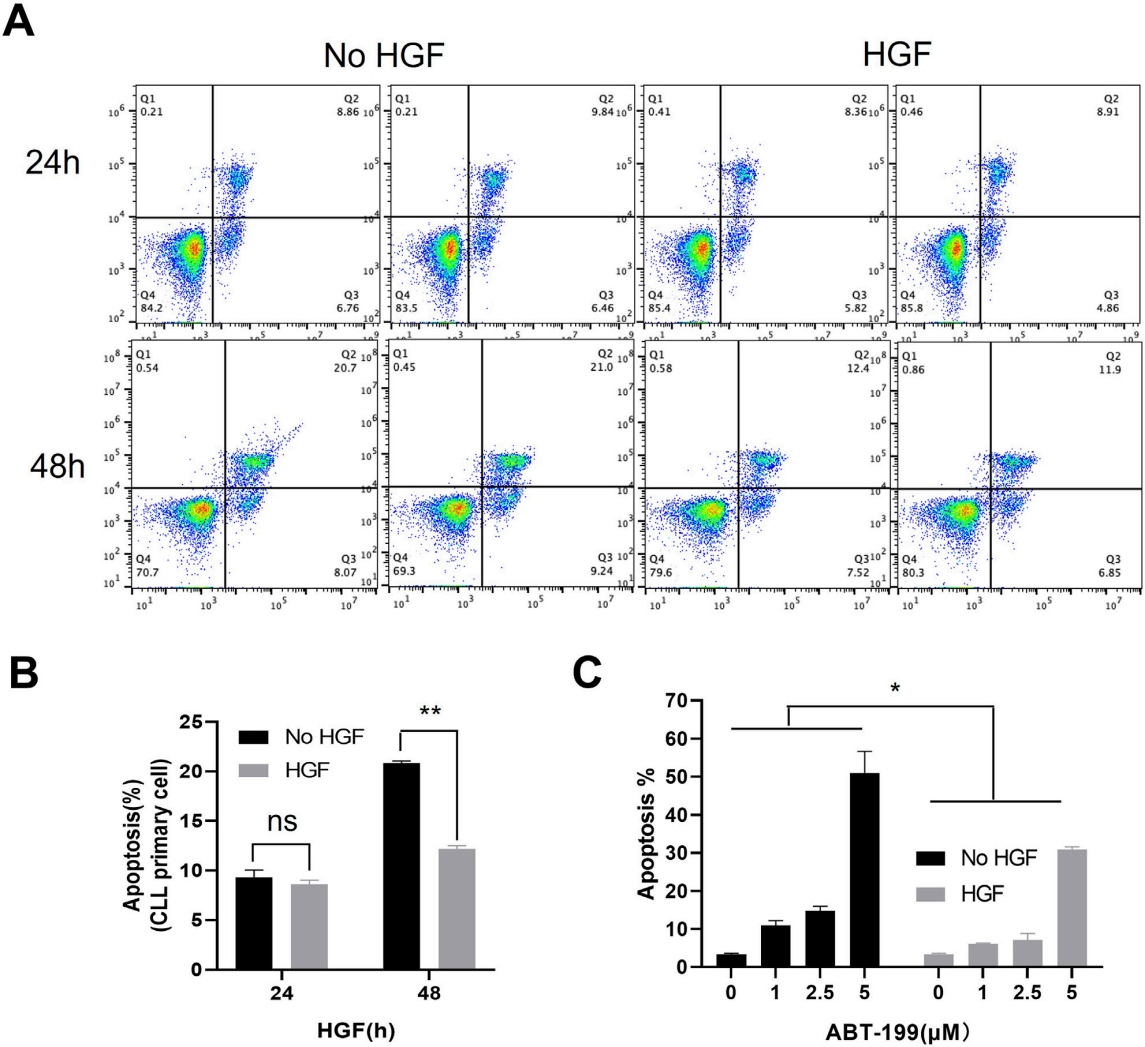
HGF enhances CLL cells survival and resistance to ABT-199. **(A)** Flow cytometry scatter plot for primary CLL cells treated with 100 ng/ml HGF, cell death was assessed by FACS after 24 or 48 h of treatments. **(B)** The percentage of apoptotic cells from **(A)** is shown. **(C)** MEC-1 cells were treated with different concentrations of ABT-199 for 48 h, and the percentage of apoptotic cells was analyzed by FACS. Statistical analysis was performed using GraphPad Prism 8.0 software and t-test. *p < 0.05, **p < 0.01, ***p < 0.001 indicate statistically significant differences between the HGF-treated group and the control group (untreated group). Non-significant results are denoted as “ns”.

To investigate the potential role of HGF in CLL, we first evaluated the effect of HGF on spontaneous apoptosis of primary CLL cells cultured *in vitro*. We observed that HGF treatment did not significantly affect spontaneous apoptosis within the first 24 h. However, a pronounced anti-apoptotic effect was evident after 48 h treatment (Figures 1A, B). Next, we examined the impact of HGF on ABT-199-induced apoptosis in the CLL cell line MEC-1. HGF treatment significantly enhanced the resistance of CLL cell line MEC-1 cells to ABT-199-induced apoptosis, indicating that HGF confers drug resistance in CLL cells. These findings suggest that HGF plays a critical role in promoting CLL cell survival and inhibiting apoptosis.

### 3.2 HGF activates multiple signaling pathways and upregulates anti-apoptotic proteins

**FIGURE 2:**
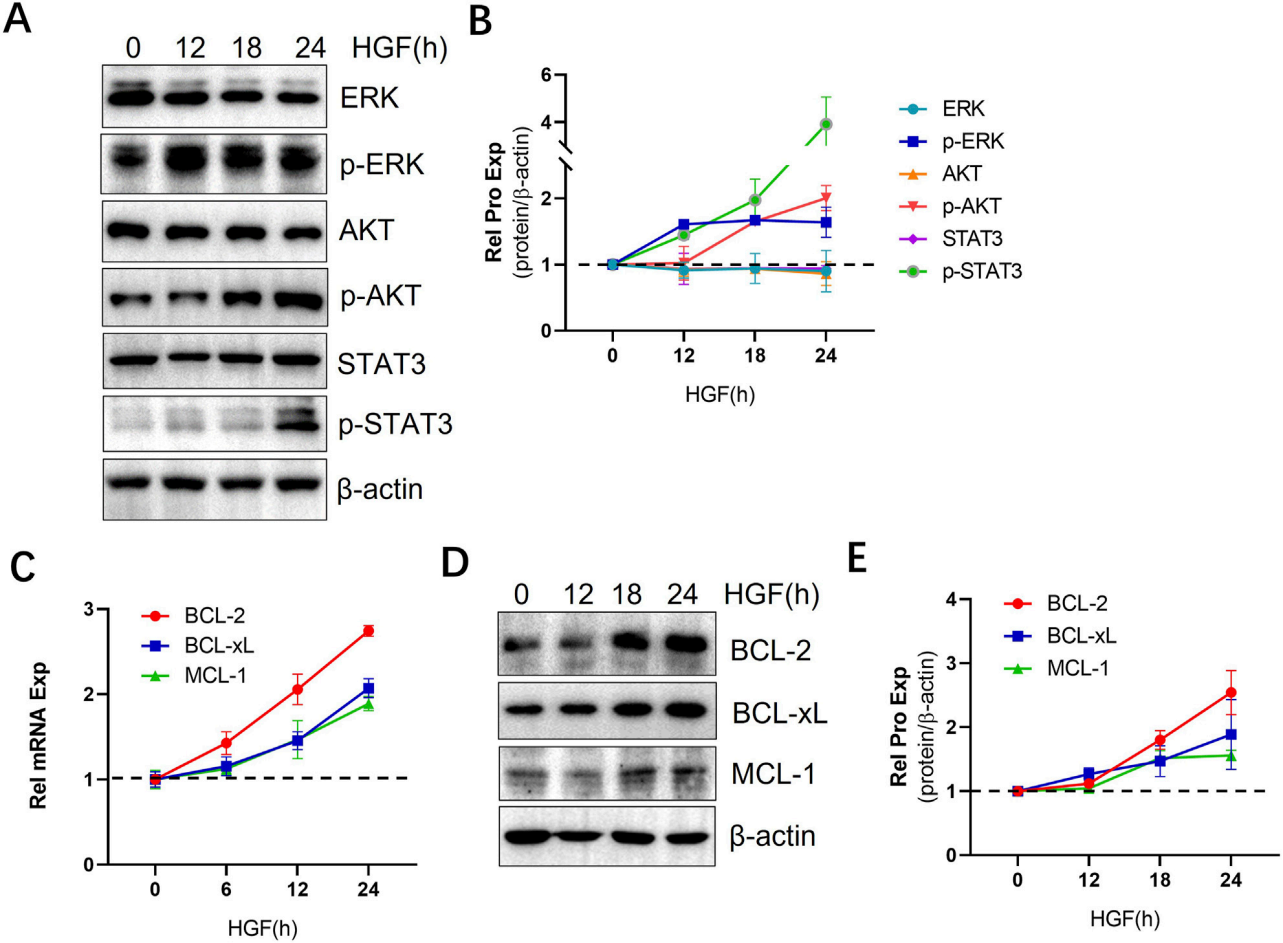
HGF activates multiple signaling pathways and upregulates anti-apoptotic proteins. **(A)** MEC-1 cells were treated with 100 ng/ml HGF and collected at different time points, WB was performed to assess the protein expression levels of AKT, p-AKT, ERK, p-ERK, STAT3, and p-STAT3. **(C)** The mRNA transcription levels and **(D)** protein expression of BCL-2 family anti-apoptotic proteins were analyzed using qPCR and WB, respectively. Relative gene expression was calculated based on threshold cycle (Ct) values and normalized to the internal control β-actin using the 2^ΔΔ^ Ct method. All the experiment were conducted in triplicate and repeated at least three times. β-actin served as the internal reference protein. **(B, E)** Quantitative analysis of the protein bands in **(A, D)** was performed using ImageJ software. The intensity of each protein bands was normalized to β-actin, and the data shown are representative images from three independent experiments.

HGF exerts its biological functions by triggering a cascade of downstream signaling pathways through its receptor, c-MET, which plays a significant role in various types of cancers (Brown, 2016; Hofbauer et al., 2010; Giannoni et al., 2011; Cascone et al., 2017; Ogunwobi et al., 2013; Ono et al., 2006). Given the crucial involvement of the AKT, ERK, and STAT3 signaling pathways in CLL cell survival, proliferation, and resistance to apoptosis (Xiao et al., 2001), we investigated the impact of HGF on the activation of these pro-survival signaling pathways in CLL cells. Our results demonstrated that HGF activates the AKT, ERK, and STAT3 signaling pathways (Figures 2A, B). The sustained activation of these pathways promotes the expression of anti-apoptotic genes (Hatano and Brenner, 2001), among these, members of the BCL-2 family are crucial regulators of apoptosis at the mitochondrial and ER membrane levels (Kapoor et al., 2020). In CLL cells, the anti-apoptotic proteins from the BCL-2 family are essential for cell survival and resistance to apoptosis (Vogler et al., 2009). Therefore, we examined the effect of HGF on the expression of pro-apoptotic proteins in CLL cells. Our findings revealed that HGF significantly upregulates the transcription and expression of anti-apoptotic proteins, including BCL-2, BCL-xL, and MCL-1, in MEC-1 cell line (Figures 2C–E). Consistent with the findings in MEC-1 cells, HGF similarly activated ERK, AKT and STAT3 signaling pathway and upregulated the expression of anti-apoptotic BCL-2 family proteins in primary CLL cells (Supplementary Figure S1A, B). These results provide a mechanistic explanation for the pro-survival and anti-apoptotic effects of HGF in CLL cells.

### 3.3 Construction of c-MET-shRNA lentivirus

**FIGURE 3:**
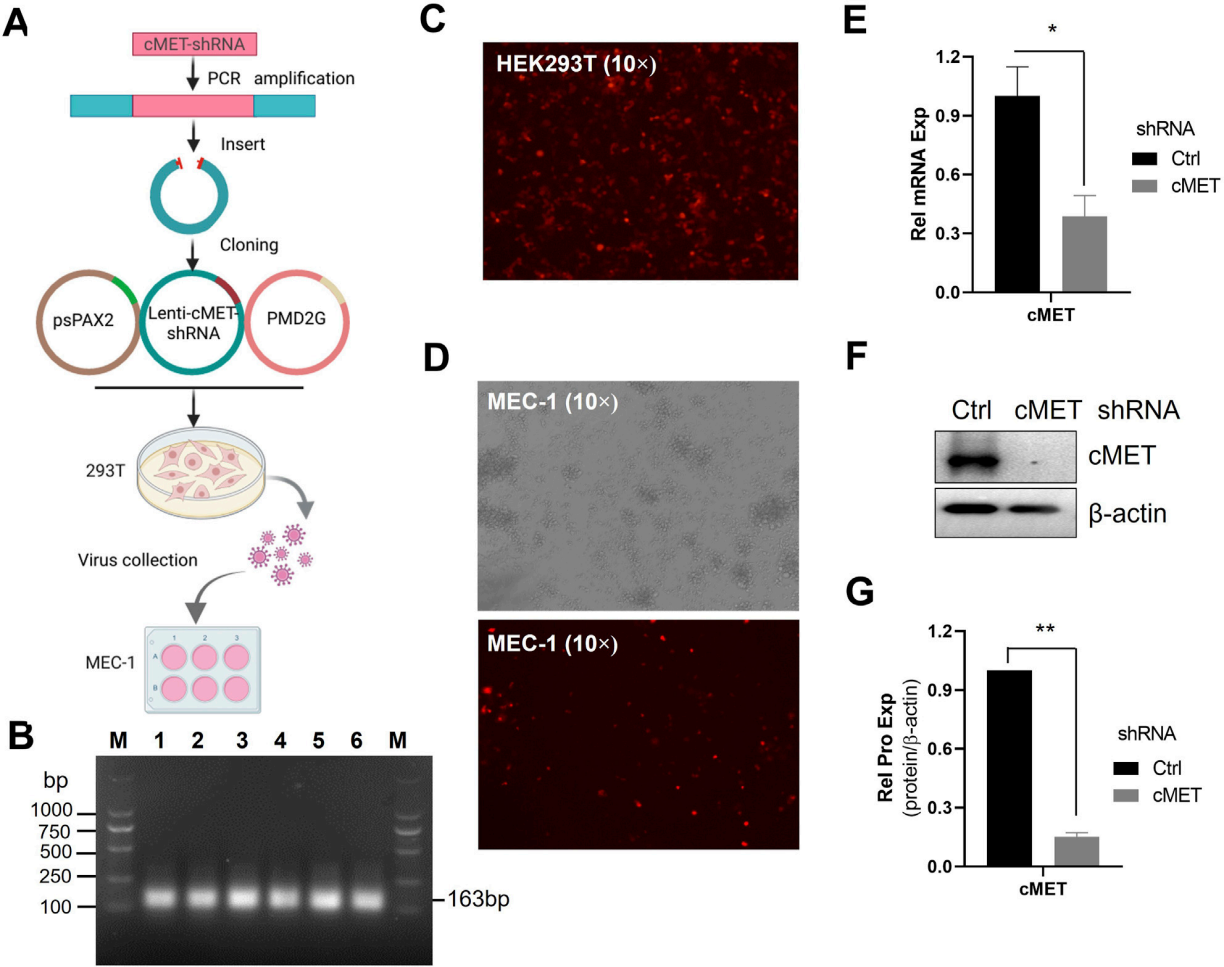
The construction of c-MET-shRNA plasmids. **(A)** Schematic flowchart of acquisition of c-MET-shRNA lentiviral particles. **(B)** PCR amplification of the c-MET-shRNA fragment. **(C)** Immunofluorescence microscopy analysis of transfection efficiency in HEK293T cells transfected with c-MET-shRNA vector plasmid and packaging plasmids. **(D)** Immunofluorescence microscopy analysis of c-MET-shRNA expression in MEC-1 cells following doxycycline induction. **(E)** RT-qPCR analysis of c-MET mRNA expression levels in MEC-1 cells infected with c-MET-shRNA lentivirus. **(F)** WB analysis of c-MET protein expression. **(G)** Quantitative analysis of the protein bands in **(F)** using ImageJ software. Band intensities were normalized to internal control β-actin.

Given the critical role of HGF in CLL cell survival, we next investigated the impact of blocking HGF-mediated signaling by targeting its receptor c-MET. To achieve this, we employed lentivirus-mediated shRNA technology to deplete c-MET, the receptor for HGF (Figure 3A). The shRNA sequences targeting c-MET were successfully amplified (Figure 3B). Subsequently, the shRNA was inserted into the Lenti-TRE-TurboRFP-miR-shRNA-zeocin vector plasmid at the XhoI and EcoRI multiple cloning sites. Positive clones were selected, and the constructed plasmid was validated by sequencing. The c-MET-shRNA plasmids, along with the packaging plasmid psPAX2 and the envelope plasmid pMD2.G, were co-transfected into HEK293T cells to generate lentiviral particles (Figure 3A). Transfection efficiency was assessed by red fluorescent protein expression under fluorescence microscopy (Figure 3C). To induce the expression of c-MET-shRNA, MEC-1 cells infected with lentiviral particles were treated with doxycycline. The results demonstrated that the majority of MEC-1 cells exhibited red immunofluorescence, indicating high transfection efficiency (Figure 3D). Three distinct shRNAs were evaluated, and all of which showed robust knockdown efficacy in MEC-1 cells (Figures 3E–G; Supplementary Figure S2). shRNA 1#, identified as the most potent, was selected for further functional experiments. The expression of c-MET was significantly suppressed at both the mRNA and protein levels after 72 h Dox induction suggesting high knockdown efficiency (Figures 3E–G). This established a reliable model for subsequent functional studies.

### 3.4 Knockdown of c-MET inhibits pro-survival signal and induces cell apoptosis

**FIGURE 4:**
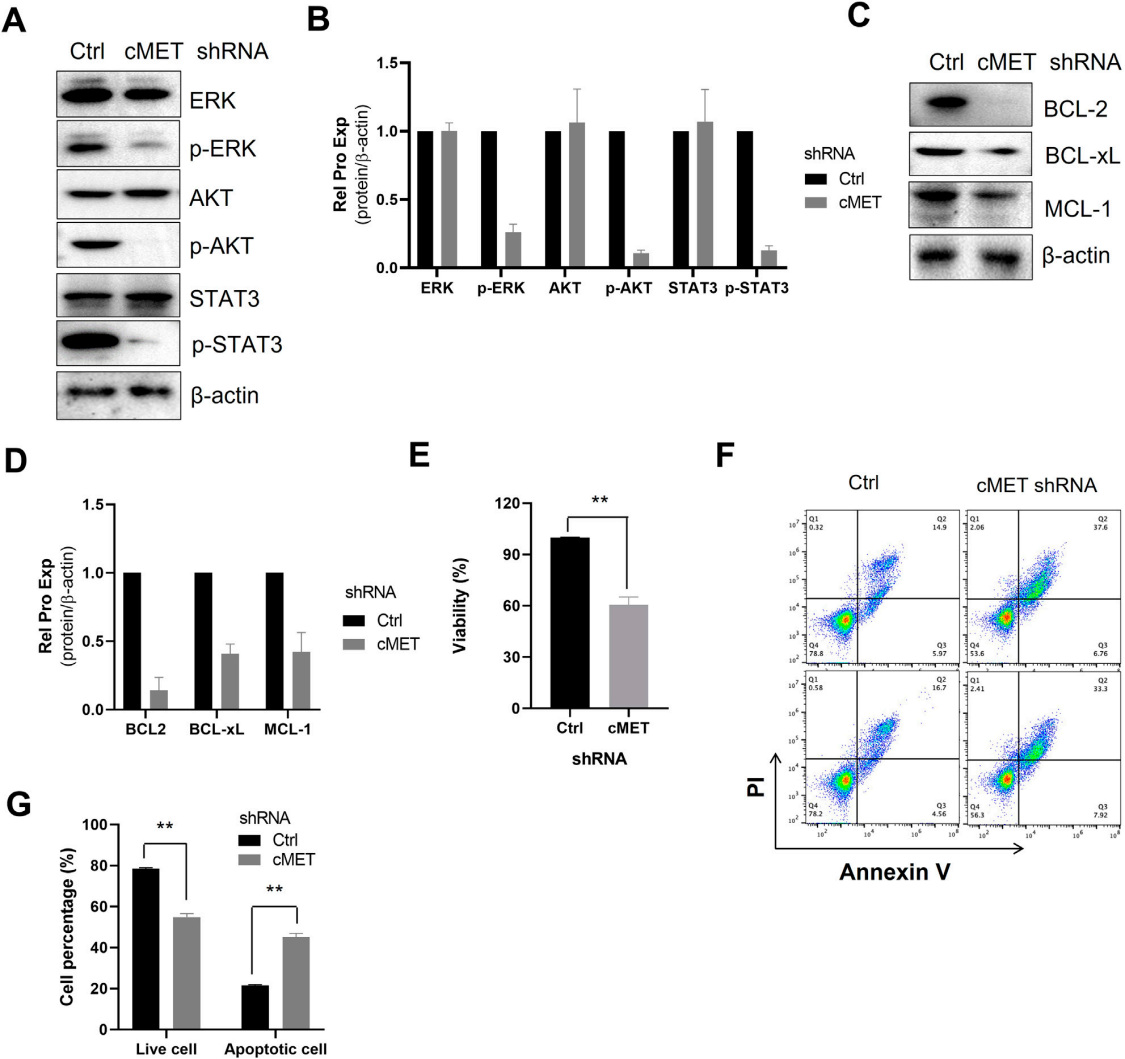
Knockdown of c-MET inhibits pro-survival signal and induces cell apoptosis. **(A)** WB detection of the expression of AKT, p-AKT, ERK, p-ERK, STAT3, p-STAT3 signaling proteins in MEC-1 cells. **(C)** WB analysis of the expression of anti-apoptotic proteins BCL-2, BCL-xL, and MCL-1 expression. β-actin was used as the internal reference protein. The data shown are representative images from two independent experiments. **(B, D)** Quantitative analysis of the protein bands in **(A, C)** using ImageJ software. Band intensities were normalized to internal control β-actin. **(E)** Cell proliferation was assessed using the MTS assay, with two sample replicates and three experimental replicates. **(F)** Flow cytometry scatter plots of cells treated with c-MET-shRNA. **(G)** The percentage of live cells and apoptotic cells relative to the total cell population. Statistical significance was determined using GraphPad Prism 8.0 software and the t-test, *p < 0.05, **p < 0.01, ***p < 0.001 indicate statistically significant differences between the c-MET-shRNA treated group and the untreated group. Non-significant results are denoted as “ns”.

Next, we investigated the impact of c-MET knockdown on pro-survival signaling in MEC-1 cells. Silencing c-MET significantly suppressed the phosphorylation of key survival-related signaling molecules, including AKT, ERK, and STAT3 (Figures 4A, B) and reduced the expression of anti-apoptotic BCL-2 family proteins (e.g., BCL-2, BCL-xL and MCL-1) (Figures 4C, D). Functionally, c-MET depletion markedly decreased MEC-1 cell viability (Figure 4E) and induced apoptosis, as evidenced by increased Annexin V/PI staining (Figures 4F, G). Together, these data indicate that c-MET is critical for maintaining pro-survival signaling and anti-apoptotic protein expression in CLL cells.

### 3.5 Targeting the HGF/c-MET axis promotes cell apoptosis in CLL

**FIGURE 5:**
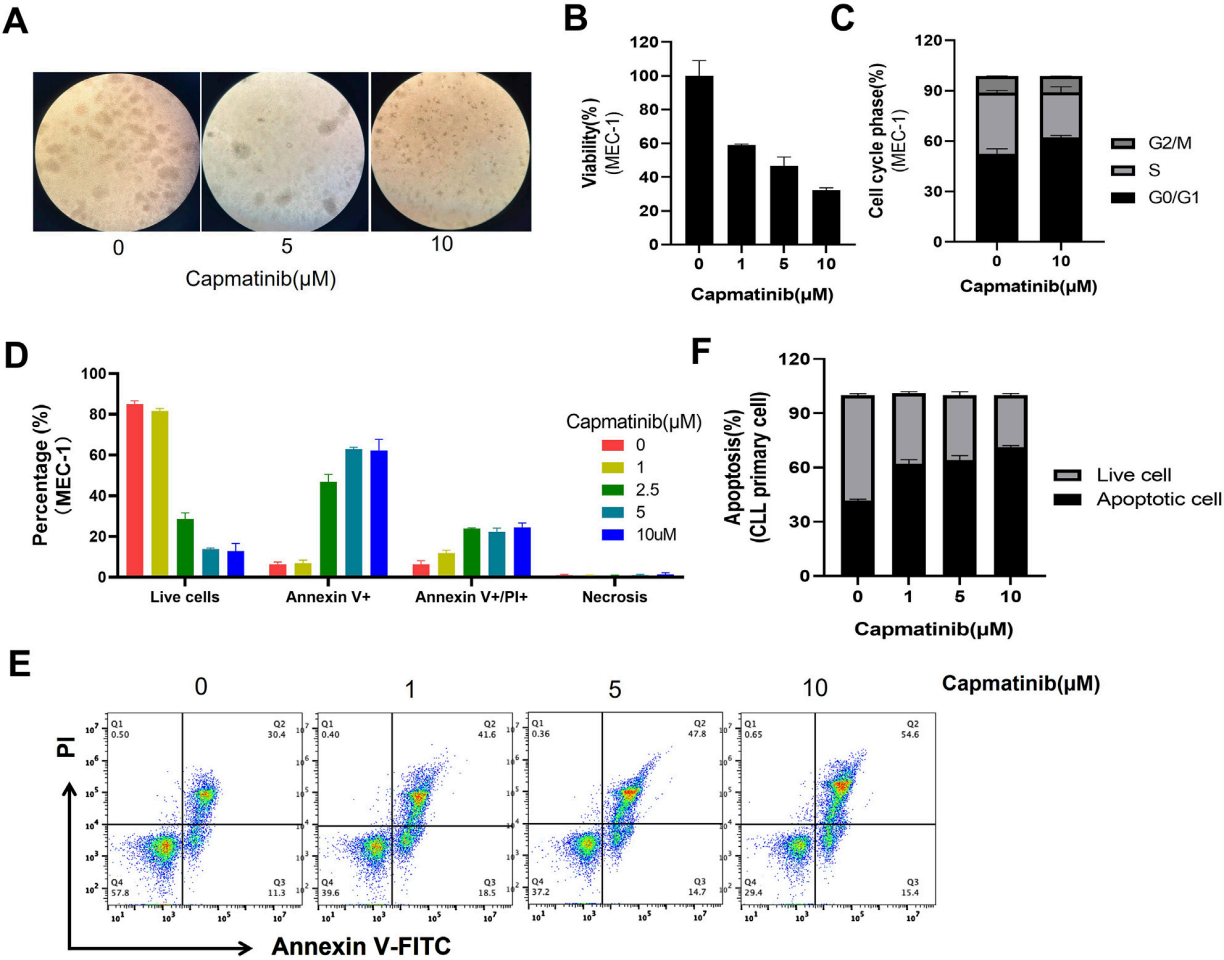
Capmatinib inhibits cell proliferation and induces apoptosis. **(A)** MEC-1 cells were treated with different concentrations of capmatinib for 48 h, and the cell viability was observed using microscopy. **(B)** Cell viability was measured using MTS assay. **(C)** The percentage of cells in different cell cycle stages. Date are representatives of three independent experiments, with results expressed as mean ± standard deviation. **(D)** The graph shows the percentage of live and apoptotic MEC-1 cells treated with different concentrations of capmatinib for 72 h. Experiments were performed in triplicate and repeated at least three times. **(E)** Flow cytometry scatter plots showing apoptosis in primary CLL cells treated with capmatinib at different doses. **(F)** The percentage of live cells and apoptotic cells in **(E)** is shown.

Given the positive regulatory role of HGF/c-MET axis in multiple pro-survival signals, and the observation that knockdown of c-MET reverses these effects, the HGF/c-MET axis emerges as a promising therapeutic target in CLL. Agents targeting HGF/c-MET, including small molecules such as crizotinib, tivantinib and cabozantinib, as well as the antibodies like rilotumumab and onartuzumab, have demonstrated efficacy in various cancers (Moosavi et al., 2021). This highlights the potential of exploring agents that block the HGF/c-MET pathway in CLL. Capmatinib, an effective, orally active, selective, and ATP-competitive c-MET kinase inhibitor, was selected for further investigation. We evaluated the effects of capmatinib on the proliferation and apoptosis in MEC-1 or EHEB cells. Our findings revealed that capmatinib effectively inhibited cell proliferation in a dose-dependent manner (Figures 5A, B; Supplementary Figure S3A), arrested the cell cycle at G0/G1 phase, and significantly increased apoptosis in MEC-1 cells (Figures 5C, D). Similarly, capmatinib promoted apoptosis in primary CLL cells cultured *in vitro* (Figures 5E, F). Notably, HGF supplementation substantially attenuated capmatinib-induced apoptosis in primary CLL cells suggesting a specific functional role of the HGF/c-MET axis in this context (Supplementary Figure S3B). Collectively, these results demonstrate that targeting the HGF/c-MET axis with the inhibitor capmatinib exerts potent anti-proliferative, cell cycle-arresting, and pro-apoptotic effects in CLL, underscoring its therapeutic potential.

### 3.6 The effects of blocking the HGF/c-MET axis on pro-survival signals in CLL cells

**FIGURE 6:**
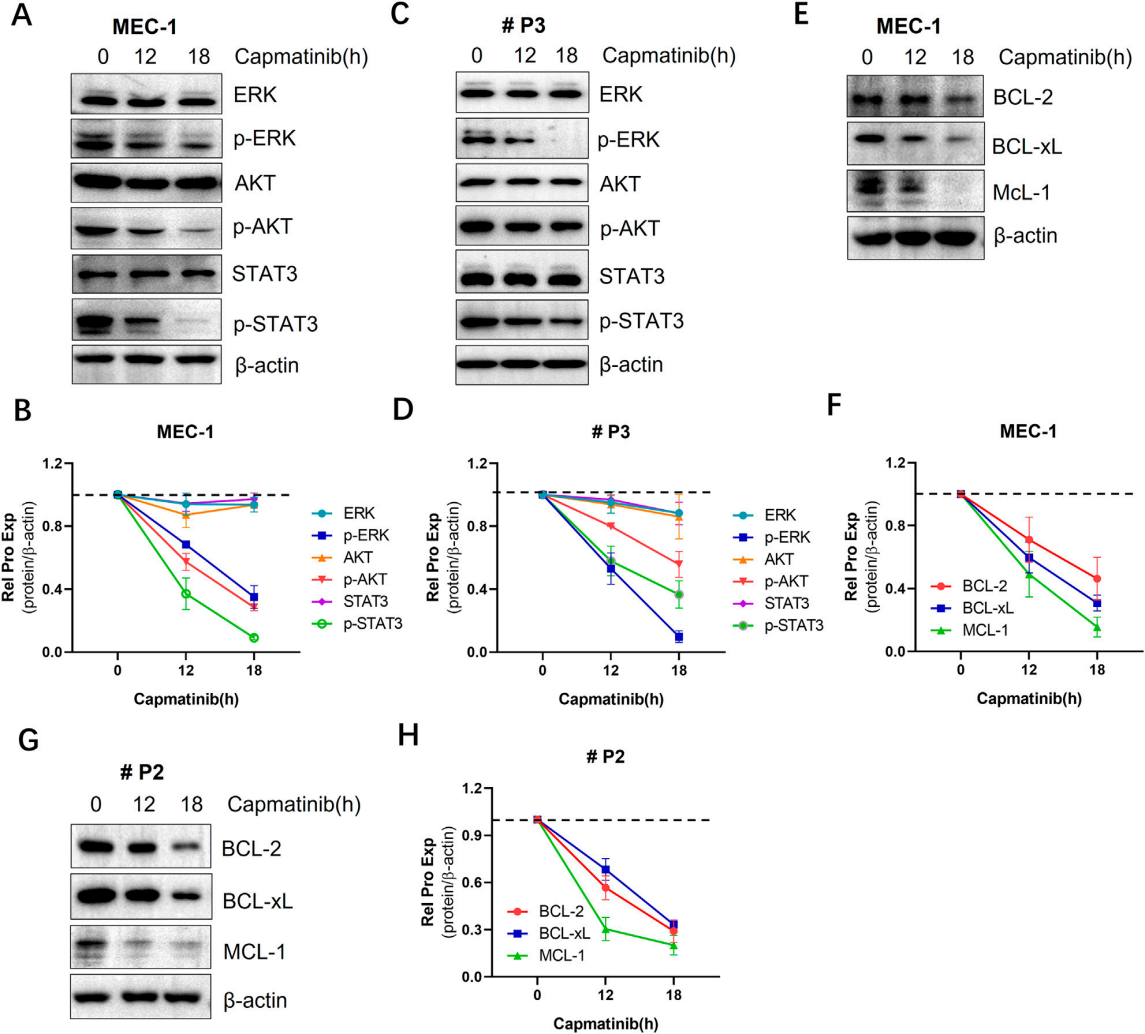
The effects of blocking the HGF/c-MET axis cell pro-survival signals in CLL cells. **(A)** WB analysis of the activation status of AKT, ERK, andSTAT3 signaling proteins in MEC-1 cells. **(C)** WB analysis of the same proteins in primary CLL cells. **(E)** WB detection of the expression levels of anti-apoptotic proteins BCL-2, BCL-xL, and MCL-1 in MEC-1 cells. **(G)** WB detection of the same anti-apoptotic proteins in primary CLL patient cells. β-actin was used as an internal reference protein for normalization. The data shown are representative images from two independent experiments. **(B, D, F, H)** Quantitative analysis of the protein bands in **(A, C, E, G)** was performed using ImageJ software. Band intensities were normalized to internal control β-actin.

The activation of the HGF/c-Met triggers a series of downstream signal pathways, playing a critical role in promoting cell survival. To investigate the impact of blocking this pathway, the c-MET inhibitor capmatinib was used. We found that capmatinib suppressed c-MET phosphorylation, and inhibited the activation of AKT, ERK and STAT3 signaling pathways (Figure 6A, B; Supplementary Figure S3C). Additionally, the expression of anti-apoptotic proteins from the BCL-2 family was markedly reduced (Figures 6E, F). Similar results were observed in primary CLL cells (Figures 6C, D, G, H).

### 3.7 Capmatinib synergizes ABT-199 to induce CLL cell apoptosis

Combination therapy is widely recognized as a promising strategy to overcome drug resistance in CLL (Cui et al., 2024; Yue et al., 2020; Chen et al., 2019a). Given the potent pro-apoptotic effects of c-MET inhibitors, we evaluated the combination of capmatinib and ABT-199 (also known as venetoclax), a highly selective and effective BCL-2 inhibitor that has shown remarkable efficacy in treating CLL (Lasica and Anderson, 2021). To assess the synergistic effects, MEC-1 cells were treated with 10 µM capmatinib in combination with 1, 2.5, and 5 μM ABT-199, while primary CLL cells were treated with 10 µM capmatinib and 0.0001, 0.001, 0.01, and 0.1 µM ABT-199. After 48 h, the cells were collected and stained with Annexin-V-FITC/PI assay kit. Apoptosis was quantified by flow cytometry. The results demonstrated that capmatinib significantly enhanced the pro-apoptotic effect of ABT-199 in both MEC-1 cells and primary CLL cells. To further validate these findings at molecular level, caspase-3 activation and PARP cleavage were assessed. The combination treatment markedly increased caspase-3 activation and PARP cleavage, indicating enhanced apoptotic signaling. These findings suggest that capmatinib has the potential to overcome ABT-199 resistance in clinical settings, highlighting its promise as a combination therapy for CLL.

## 4 Discussion

Although recent therapeutic advancements have significantly improved the survival of patients with CLL, the disease remains incurable. Therefore, continued efforts to explore its underlying mechanisms and develop effective treatment strategies are essential. Aberrant activation of the HGF/c-MET axis has been shown to play a critical role in the development and progression of various human cancers (Catenacci et al., 2017; Heo et al., 2017; Gayyed et al., 2015; Peltola et al., 2017) and is often associated with poor clinical outcome and drug resistance (Mahtouk et al., 2010; Guo et al., 2020). While the role of HGF/c-MET activation has been extensively studies and therapeutically targeted in solid tumors (Matsumoto et al., 2017), its involvement in hematological malignancies, including CLL, remains poorly understood. In this study, we demonstrate that HGF/c-MET axis significantly enhances the survival and anti-apoptotic capacity of CLL cells by regulating multiple pro-survival mechanisms. Conversely, knockdown of the c-MET gene or inhibition of HGF/c-MET signaling markedly attenuates these effects. These findings suggest that targeting the HGF/c-MET pathway could represent a promising novel therapeutic approach for CLL.

The survival of CLL cells is highly dependent on their microenvironment, where interactions with stromal cells, monocytes, and T cells receives various stimulatory signals. Within the microenvironment, multiple cytokines promote CLL cells survival and contribute to drug resistance (Chiorazzi et al., 2021; Chen et al., 2022; Chen et al., 2019b). HGF is a multifunctional growth factor known to induce diverse physiological responses in various cell types. In this study, we demonstrated that cytokine HGF enhances the ability of CLL cells to resist both natural and ABT-199-induced apoptosis *in vitro* (Figures 1A–C), suggesting its critical role in CLL cell survival and anti-apoptosis. Previous studies have reported that the elevated plasma HGF levels serve as a prognostic factor in AML patients (Verstovsek et al., 2001) Similarly, patients with lymphoma exhibit increased HGF levels in their blood, bone marrow, plasma, and pleural fluid (Tjin et al., 2006). In CLL patients, serum HGF level are significantly higher compared to healthy controls (Eksioglu-Demiralp et al., 2011), and high HGF levels are associated with survival benefit for CLL cells. However, the underlying mechanisms remain poorly understood. In this study, we demonstrated that HGF regulates multiple pro-survival signaling pathways. Specifically, HGF activates the AKT, ERK, and STAT3 pathways (Figures 2A, B; Supplementary Figure S1A, B), which are crucial for CLL cell survival, proliferation, and resistance to apoptosis and drug treatment (Xiao et al., 2001). These findings align with previous observations in other cancer types. Although the precise mechanisms by which HGF activates these pathways are not fully elucidated, it is hypothesized that c-MET, a high-affinity tyrosine kinase receptor for HGF, plays a central role. The intracellular domain of the c-MET protein includes a proximal membrane domain, a kinase catalytic domain, and a C-terminal domain. The C-terminal domain contains a unique binding site where phosphorylation of Tyr1349 and Tyr1356 recruits various signal transduction proteins and adapter proteins, thereby activating multiple downstream signaling pathways (Rocci et al., 2014; Birchmeier et al., 2003). The sustained activation of these pro-survival signaling pathways promotes the expression of anti-apoptotic proteins (Hatano and Brenner, 2001). We found that HGF significantly upregulates the expression of anti-apoptotic proteins, including BCL-2, BCL-xL, and MCL-1, at both mRNA and protein levels in CLL cells (Figures 2C–E). In addition, HGF also upregulates the mRNA expression of genes such as CD11a, BCR, CCL2, CCL3, CCL17, CCL22, CD38, c-MET, TACI, BCMA, NOTCH1, CCR7, CD44, CXCR3 CXCR4 and CXCR5 (Supplementary Figure S4). These genes are closely associated with to CLL cell adhesion, migration, and tissue homing. Collectively, these findings provide mechanistic explanation for the pro-survival function of the HGF/c-MET axis in CLL.

The HGF/c-MET axis is widely present in various cell types and plays a crucial role in the physiological regulation of tissue and organ growth and development. However, dysregulation of this axis is frequently associated with carcinogenesis. For example, in non-small cell lung adenocarcinoma (NSCLC), c-MET overexpression is observed in approximately 20%–25% of clinical cases. Similarly, studies have reported elevated c-MET mRNA expression in AML THP1 cells (Giannoni et al., 2014). Additionally, c-MET is often overexpressed in DLBCL (Tjin et al., 2006; Jücker et al., 1994). In blood cancers, nurse-like cells (NLCs) expressing c-MET exhibit aggressive characteristics (Giannoni et al., 2014; Rocci et al., 2014). Notably, cells resembling NLCs in the bone marrow (BM) and lymph nodes of CLL patients are frequently c-MET positive (Giannoni et al., 2014). In this study, we employed lentivirus-mediated shRNA technology to knock down c-MET expression and assess its effects on cell survival and anti-apoptotic mechanisms. Our results demonstrated that c-MET depletion significantly inhibits the activation of the AKT, ERK, and STAT3 signaling pathways (Figures 4A, B) and reduces the expression of anti-apoptotic proteins in the BCL-2 family. (Figures 4C, D), Consequently, c-MET knockdown markedly suppressed cell proliferation and promoted apoptosis (Figures 4E–G). Together, these findings confirm the critical role of the HGF/c-MET axis in CLL cell survival and anti-apoptosis, highlighting its potential as a therapeutic target in CLL treatment.

c-MET signaling is thought to play a crucial role in promoting the development and maintenance of the tumor microenvironment, facilitating immune evasion by tumor cells and enabling them to escape T-cell-mediated killing (Dempke et al., 2022; Zambelli et al., 2021). Additionally, abnormal activation of c-MET tyrosine kinase is a key driver of resistance to targeted therapies (Rivas et al., 2022). As a result, the HGF/c-MET pathway has emerged as a significant therapeutic target in various cancers (Lee et al., 2021; Hu et al., 2024). Studies have demonstrated that knockdown of c-MET induces apoptosis in multidrug-resistant cancer cell lines, underscoring its potential as a promising therapeutic target (Hung et al., 2015). Agents targeting the HGF/c-MET axis have shown clinical efficacy across multiple tumor types (Moosavi et al., 2021). For example, targeting c-MET has been investigated as a potential treatment strategy for small cell lung cancer (Friedlaender et al., 2020). A phase II study with the c-MET inhibitor tivantinib in patients with relapsed/refractory multiple myeloma (MM) has been recently reported (Baljevic et al., 2017). Moreover, treatment with crizotinib was shown to reduce colony formation in HGF-expressing primary AML samples (Kentsis et al., 2012). Furthermore, crizotinib demonstrated efficacy in suppressing the growth of primary effusion lymphoma (PEL) both *in vitro* and *in vivo*, as evidenced by a pre-clinical NOD/SCID mouse model (Dai et al., 2015).

**FIGURE 7:**
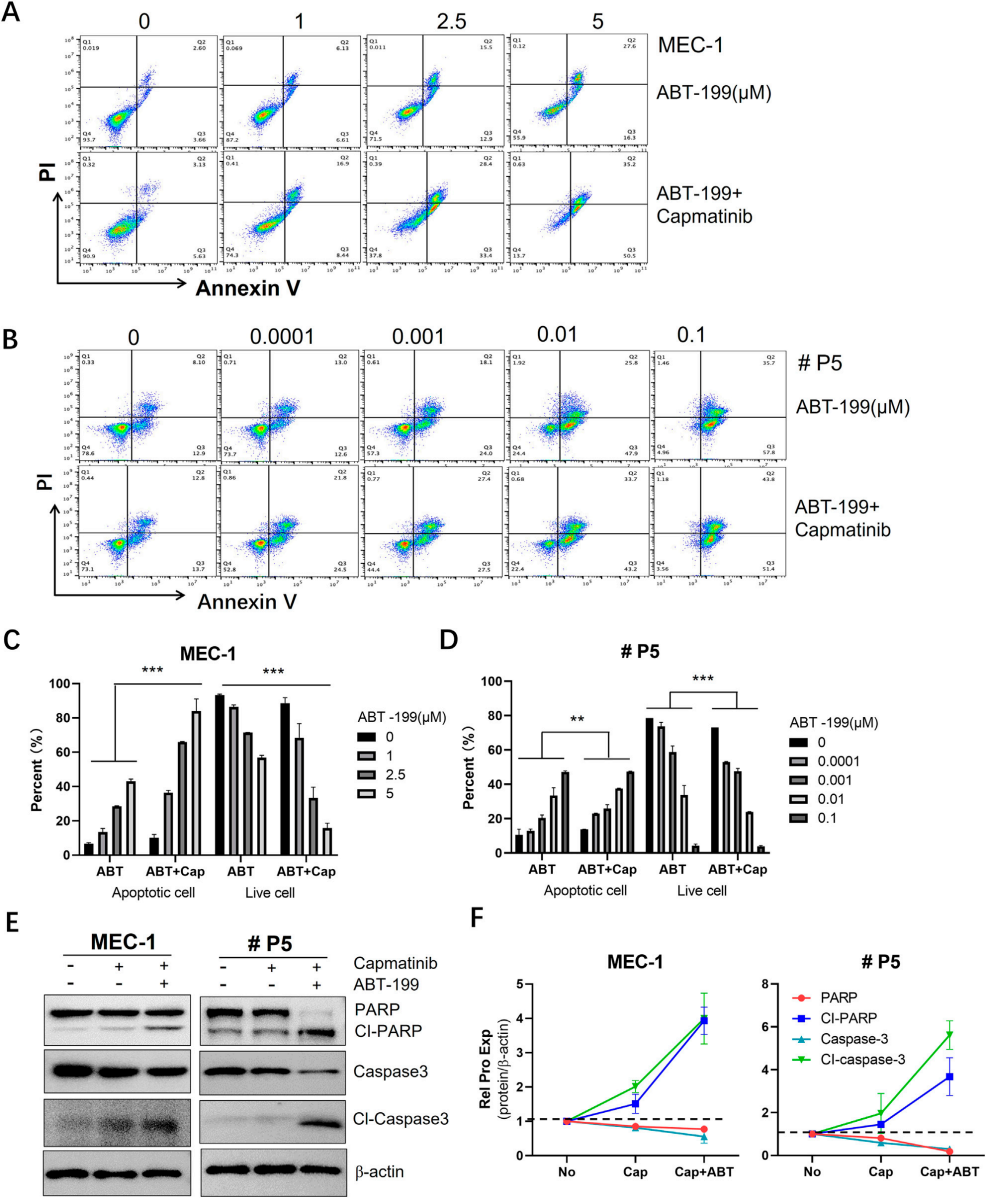
The synergistic effect of capmatinib and ABT-199 on inducing apoptosis in CLL cells. Flow cytometry scatter plots of **(A)** MEC-1 cells and **(B)** primary CLL cells treated with ABT-199 alone or combing with capmatinib. **(C, D)** The quantification analysis of apoptotic cells and live cells in **(A, B)**. The graphs show representative of three independent experiments, with results expressed as mean ± standard deviation. **(E)** WB analysis of the protein expression of PARP and caspase-3 in MEC-1 cells and primary CLL cells. **(F)** Quantitative analysis of protein bands in **(E)**. Statistical significance was determined using GraphPad Prism 8.0 software and the two-way ANOVA. *p < 0.05, **p < 0.01, ***p < 0.001 indicate that statistically significant differences between capmatinib-treated group and untreated group. Non-significant results are denoted as “ns”.

Although studies on the activation of the c-MET receptor in hematological diseases are relatively limited, emerging evidence suggests that the interaction between c-MET and its HGF ligand may promote the expansion of the leukemic clones. Therefore, targeting c-MET could represent a promising therapeutic strategy for leukemia. Consistent with this notion, our findings demonstrate that targeting the HGF/c-MET axis with the inhibitor capmatinib is a viable approach for CLL treatment, offering potential benefits to CLL patients. Notably, capmatinib has already shown significant antitumor activity in patients with advanced NSCLC harboring c-MET exon 14 skipping mutations, particularly in treatment-naïve individuals (Wolf et al., 2020). In the context of CLL, our results reveal that capmatinib effectively inhibits cell proliferation, induces cell cycle arrest, and promotes apoptosis (Figures 5A–F). These effects are mediated through the suppression of multiple pro-survival signaling pathways and the downregulation of anti-apoptotic proteins, highlighting its potent anti-tumor activity (Figures 6A–I). While ABT-199 (venetoclax) has demonstrated remarkable efficacy in CLL treatment (Lasica and Anderson, 2021), its therapeutic impact is often compromised by the overexpression of BCL-2 and other anti-apoptotic proteins such as MCL-1. Combination therapy has emerged as a strategic approach to overcome such drug resistance. In this study, we provide evidence that capmatinib enhances the pro-apoptotic effects of ABT-199, suggesting the potential for combination therapy in CLL treatment (Figure 7).

**FIGURE 8:**
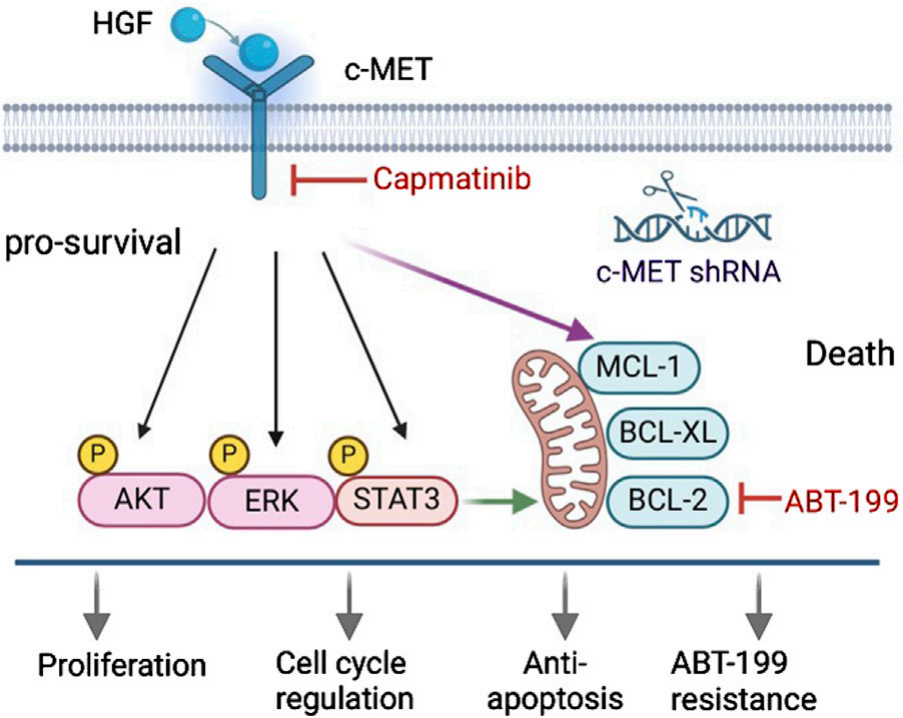
Targeting the HGF/c-MET axis suppresses pro-survival signaling and sensitizes CLL cells to ABT-199-induced apoptosis. The HGF/c-MET axis promotes CLL cells survival by activating AKT, ERK, and STAT3 pro-survival pathways and upregulating BCL-2 family anti-apoptotic proteins, which collectively confer resistance to ABT-199-induced apoptosis. Genetic silencing of c-MET or pharmacological blockade of HGF/c-MET signaling via capmatinib disrupts these survival mechanisms, suppresses proliferation, and restores sensitivity to ABT-199-induced cell death.

The interaction of HGF with c-MET triggers the activation of multiple downstream signaling pathways, including AKT, ERK, and STAT3, leading to the expression of various anti-apoptotic proteins such as BCL-2, BCL-xL and MCL-1. Consequently, this enhances the survival capability of CLL cells. Conversely, knockdown of the c-MET gene or inhibition of the HGF/c-MET pathway using specific inhibitors significantly attenuates these pro-survival mechanisms, thereby promoting cell apoptosis (Figure 8). In summary, this study demonstrates that the HGF/c-MET pathway plays a critical role in CLL cell survival. It provides mechanistic insights into how this signaling axis influences CLL cell survival and highlights potential therapeutic targets for further investigation in CLL.

## Data Availability

The original contributions presented in the study are included in the article/Supplementary Material, further inquiries can be directed to the corresponding author.
