# Supplementary material for: HGF/c-MET axis contributes to CLL cell survival by regulating multiple mechanisms making it a potential therapeutic target for CLL treatment
Source: Front Pharmacol. 2025 May 30;16:1612916. doi: 10.3389/fphar.2025.1612916 (PMC12162569; doi:10.3389/fphar.2025.1612916)
Supplement: Supplementary file 1 [file DataSheet1.docx]

**HGF/c-MET axis contributes to CLL cell survival by regulating multiple mechanisms making it a potential therapeutic target for CLL treatment**

Shihao Liang^1#^, Xiaoya Shao^2#^, Xueqiong Meng^3,4,5^, Ying Cui^1^, Chuanyue Sun^1^, Jie Sun^1^, Binghui Zhang^1^, Guomin Shen^1^, Ling Qin^6^, Haiping Yang^6^, Yixiang Chen^1,3,5*^

**Supplementary data**

**2. Materials and methods**

**Supplementary Table 1 Clinical data available for CLL patients**

| **CLL Patients** | **Sex** | **Age** | **Cytogenetics** |
| --- | --- | --- | --- |
| P1 | M | 82 | RB1(13q14) deletion, D13S25(13q14) deletion, ATM(11q22) deletion |
| P2 | M | 71 | RB1(13q14) gene, ATM (11q22.3) gene normal |
| P3 | M | 79 | TP53, chromosome 12 and 13 normal |
| P4 | M | 70 | ATM, NOTCH1, SF3B1 genes normal, p53 deletion (35%) |
| P5 | M | 92 | NOTCH1 gene p.P2138Rfs*110 frameshift mutation (33.0%), SF3B1 gene p.N626S missense mutation (4.2%), ATM gene deletion; BIRC3, BTK, MYD88, PLCG2, TP53 genes normal |
| P6 | F | 80 | p53 (17p13) gene deletion |
| P7 | M | 43 | No mutation in ATM, BTK, BIRC3, MYD88, NOTCH1, PLCG2, SF3B1 and TP53 genes, IGH/ccnd1 normal, no 17q deletion |

**Supplementary Table 2 c-MET-shRNA sequences**

| SplashRNA  ([NM_000245.4](https://www.ncbi.nlm.nih.gov/nuccore/NM_000245.4?report=GenBank)) | 97bp sequence |
| --- | --- |
| 2.2405812 | TGCTGTTGACAGTGAGCGACGAGATGAATGTGAATATGAATAGTGAAGCCACAGATGTATTCATATTCACATTCATCTCGGTGCCTACTGCCTCGGA |
| 2.008130666 | TGCTGTTGACAGTGAGCGCGACAAGTAATTTGTTGATAAATAGTGAAGCCACAGATGTATTTATCAACAAATTACTTGTCTTGCCTACTGCCTCGGA |
| 1.946416089 | TGCTGTTGACAGTGAGCGACAGGAAAATGATTATAACTAATAGTGAAGCCACAGATGTATTAGTTATAATCATTTTCCTGCTGCCTACTGCCTCGGA |

**Supplementary Table 3 PCR primers of lentiviral plasmid construction**

| **primer** | **sequences** |
| --- | --- |
| miRE-F-XhoI： | 5‘-cttaacccaacagaaggCTCGAGAAGGTATATTGCTGTTGACAGTGAGCG-3’ |
| miRE-R-EcoRI： | 5‘-ACAAGATAATTGCTCGAATTCTAGCCCCTTGAAGTCCGAGGCAGTAGGCA-3‘ |

**Supplementary Table 4** qPCR primers

| Gene | Upper primer (5' -> 3') | Lower primer (5' -> 3') |
| --- | --- | --- |
| c-MET | AGCGTCAACAGAGGGACCT | GCAGTGAACCTCCGACTGTATG |
| MCL-1 | TGCTTCGGAAACTGGACATCA | TAGCCACAAAGGCACCAAAAG |
| BCL-2 | GGTGGGGTCATGTGTGTGG | CGGTTCAGGTACTCAGTCATCC |
| BCL-xL | GAGCTGGTGGTTGACTTTCTC | TCCATCTCCGATTCAGTCCCT |
| β-actin | AAAGACCTGTACGCCAACAC | GTCATACTCCTGCTTGCTGAT |

**3. Results**

**Figure 1**


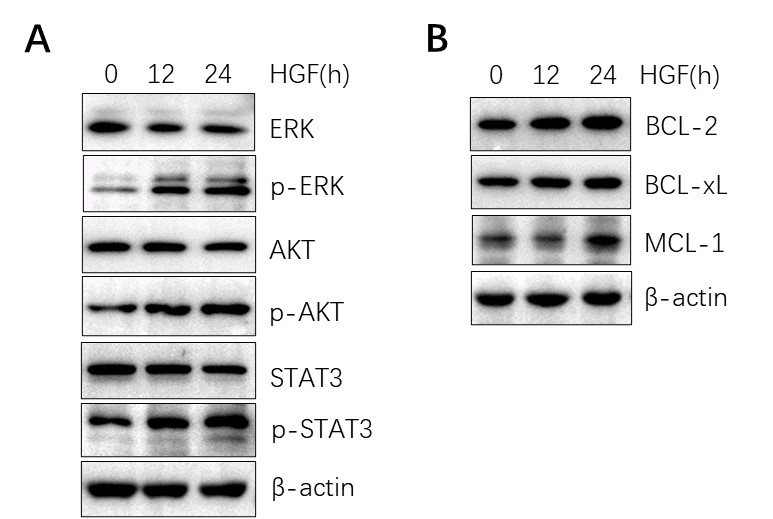


**Figure 1 HGF activates multiple signaling pathways and upregulates anti-apoptotic proteins in primary CLL cells.** (A) Primary CLL cells were treated with 100ng/ml HGF and harvested at indicated time points, WB analysis was performed to assess the protein expression levels of ERK, p-ERK, AKT, p-AKT, STAT3, and p-STAT3. (D) Protein expression of BCL-2 family anti-apoptotic proteins was analyzed by WB. β-actin was used as a loading control.

**Figure 2**


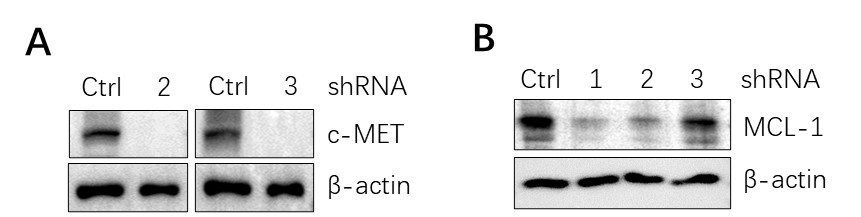


**Figure 2 Validation of c-MET-shRNA knockdown efficiency in MEC-1 cells.** (A) WB analysis of c-MET protein expression in MEC-1 cells transduced with c-MET-shRNA lentivirus. (B) WB analysis of MCL-1 protein expression in MEC-1 cells infected with c-MET-shRNA lentivirus. β-actin served as the internal control protein.

**Figure 3**


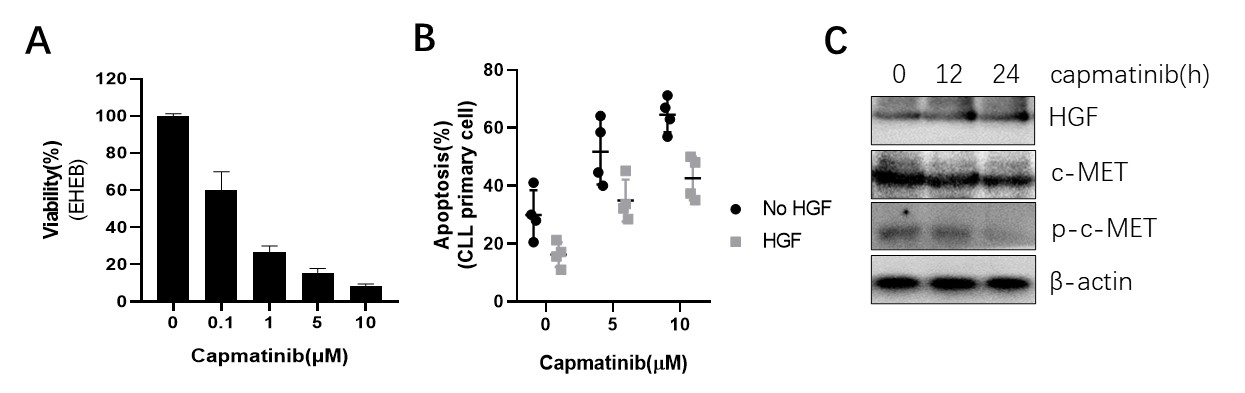


**Figure 3 Capmatinib exerts anti-proliferative and pro-apoptotic effects in CLL cells.** (A) Viability of EHEB cells treated with different concentrations of capmatinib for 48 hours, assessed via MTS assay. (B) Apoptosis in primary CLL cells (n=4) treated with different concentrations of capmatinib or capmatinib+HGF(100ng/ml) for 48 hours, was analyzed by FACS. (C) The expression levels of HGF, c-MET, and p-c-MET in primary CLL patient sample (#P2) treated with capmatinib, were detected by WB assay. β-actin served as a loading control.

**Figure 4**

**Figure 4 The mRNA expression of genes-induced by HGF.** RT-qPCR analysis of the mRNA expression of CD11a, BCR, CCL2, CCL3, CCL17, CCL22, CD38, c-MET, TACI, BCMA, NOTCH1, CCR7, CD44, CXCR3 CXCR4 and CXCR5 genes in MEC-1 cells stimulated with 100ng/ml HGF.
